# Supplementary material for: Human brain integrates both unconditional and conditional timing statistics to guide expectation and behavior
Source: PLoS Biol. 2025 Oct 23;23(10):e3003459. doi: 10.1371/journal.pbio.3003459 (PMC12561982; doi:10.1371/journal.pbio.3003459)
Supplement: S1 Text — (DOCX) [file pbio.3003459.s001.docx]

**Supplementary Methods**

In addition to the hazard function, there were other representations for predicting when an event would occur, including the original probability distribution, temporally blurred probability distribution, and probabilistically blurred probability distribution, as well as their transformation to hazard functions (Grabenhorst et al., 2019, 2021). First, although the hazard function can represent dynamic updates of temporal predictions exactly, it has been challenged to describe its continuous and complicated computations over time in the brain, and the probability distribution as the foundation of a hazard function has been proposed for temporal prediction representations. Second, uncertainty in estimating probabilities increases over time. In other words, the brain predicts a stimulus onset less precisely as time elapses. This phenomenon is termed the temporally blurred effect, where uncertainty is modeled by a Gaussian function with the standard deviation scaling with time. The equations for the temporally blurred probability distribution and hazard function are listed below.

$f_{tb}\left( t \right)= \frac{1}{\varphi t\sqrt{2\pi}}\int_{-\infty}^{\infty} f(\tau)\cdot e^{{-\left( \tau-t \right)}^{2}/ ({2\varphi}^{2}t^{2})}d\tau$ (1)

${HF}_{tb}\left( t \right)= \frac{f_{tb}\left( t \right)}{1-C_{tb}(t)}$ (2)

where *t* represents time points, ranging from 0.4 to 2 sec. The function *f_tb_* represents the temporal-blurred probability distribution of the foreperiod in a block. $t\cdot\varphi$ represents the standard deviation scaling with time. In the current study, we tested $\varphi$ from 0.15 to 0.35 and show the comparisons with the parameter of 0.21 based on previous research (Grabenhorst et al., 2019, 2021). The function $C_{tb}$ represents the accumulation of temporal-blurred probabilities up to time *t*. When $1- C_{tb}(t)$ approaches zero, ${HF}_{tb}(t)$ becomes an infinite value, and we replaced the infinite value with the maximum hazard value before time *t*. Subsequently, hazard values were normalized between 0 to1.

Third, uncertainty can also be scaled with precision, meaning that the brain predicts a stimulus onset less precisely due to the lower chance (probability) at that specific time. This is termed the probabilistically blurred effect, where uncertainty is modeled by a Gaussian function with the standard deviation scaling with probability.

$\sigma_{min}= \varphi\cdot t_{min}= \varphi\cdot0.4$ (3)

$\sigma_{max}= \varphi\cdot t_{max}= \varphi\cdot2.0$ (4)

$s\left( t \right)=\left[ 1- \frac{\left( f\left( t \right)-f_{min} \right)}{{(f}_{max}- f_{min})} \right]\cdot\left( \sigma_{max}-\sigma_{min} \right)+\sigma_{min}$ (5)

$f_{pb}\left( t \right)= \frac{1}{s(t)\sqrt{2\pi}}\int_{-\infty}^{\infty} f(\tau)\cdot e^{{-\left( \tau-t \right)}^{2}/ (2{s(t)}^{2})}d\tau$ (6)

where $\varphi$ was tested from 0.15 to 0.35 as well. The function $s$ represents how standard deviation scales with probability, where higher probabilities lead to lower standard deviations and vice versa. The function *f_pb_* represents the probabilistically blurred probability distribution of the foreperiod in a block. The transformation from *f_pb_* to the probabilistically blurred hazard function is the same as the formula (2).

**References**

Grabenhorst, M., Maloney, L. T., Poeppel, D., & Michalareas, G. (2021). Two sources of uncertainty independently modulate temporal expectancy. *Proceedings of the National Academy of Sciences*, *118*(16), e2019342118. https://doi.org/10.1073/pnas.2019342118

Grabenhorst, M., Michalareas, G., Maloney, L. T., & Poeppel, D. (2019). The anticipation of events in time. *Nature Communications*, *10*(1), 5802. https://doi.org/10.1038/s41467-019-13849-0
