# Supplementary material for: Human brain integrates both unconditional and conditional timing statistics to guide expectation and behavior
Source: PLoS Biol. 2025 Oct 23;23(10):e3003459. doi: 10.1371/journal.pbio.3003459 (PMC12561982; doi:10.1371/journal.pbio.3003459)
Supplement: S1 Table — (DOCX) [file pbio.3003459.s002.docx]

| Block 1 | Block 2 | Block 3 | Block 4 |
| --- | --- | --- | --- |
| 5.02 $\pm$ 6.29 | 3.95 $\pm$ 5.08 | 5 $\pm$ 6.04 | 4.33 $\pm$ 5.32 |

Mean $\pm$ Standard deviation.
