# Supplementary material for: Human brain integrates both unconditional and conditional timing statistics to guide expectation and behavior
Source: PLoS Biol. 2025 Oct 23;23(10):e3003459. doi: 10.1371/journal.pbio.3003459 (PMC12561982; doi:10.1371/journal.pbio.3003459)
Supplement: S2 Table — (DOCX) [file pbio.3003459.s003.docx]

| RTs | Block 1 | Block 2 | Block 3 | Block 4 |
| --- | --- | --- | --- | --- |
| FP1 | 0.237 $\pm$ 0.029 | 0.237 $\pm$ 0.030 | 0.241 $\pm$ 0.035 | 0.240 $\pm$ 0.038 |
| FP2 | 0.225 $\pm$ 0.030 | 0.240 $\pm$ 0.033 | 0.233 $\pm$ 0.033 | 0.242 $\pm$ 0.036 |
