# Supplementary material for: Human brain integrates both unconditional and conditional timing statistics to guide expectation and behavior
Source: PLoS Biol. 2025 Oct 23;23(10):e3003459. doi: 10.1371/journal.pbio.3003459 (PMC12561982; doi:10.1371/journal.pbio.3003459)
Supplement: S3 Table — (DOCX) [file pbio.3003459.s004.docx]

| Fixed effects | **AIC** | **BIC** | ***Adj R^2^*** |  |
| --- | --- | --- | --- | --- |
| RT following FP1 (false alarm removed) | | | | |
| ~ HF_U_ | -43719.0 | -43687.8 | 0.173 |  |
| RT following FP1 (false alarm & outliers removed) | | | | |
| ~ HF_U_ | -54015.2 | -53984.2 | 0.262 |  |
|  |  |  |  |  |
| RT following FP2 (false alarm removed) | | | | |
| ~ HF_U_ | -48388.5 | -48349.6 | 0.218 |  |
| ~ HF_C_ | -48335.4 | -48296.5 | 0.215 |  |
| ~ HF_U_ + HF_C_ | -48386.8 | -48340.1 | 0.218 |  |
| ~ HF_U_ + HF_C_ + HF_U_* HF_C_ | -48449.7 | -48395.1 | 0.221 |  |
| RT following FP2 ( false alarm & outliers removed) | | | | |
| ~ HF_U_ | -56512.3 | -10776.3 | 0.300 |  |
| ~ HF_C_ | -56423.5 | -10630.9 | 0.295 |  |
| ~ HF_U_ + HF_C_ | -56510.5 | -10767.9 | 0.300 |  |
| ~ HF_U_ + HF_C_ + HF_U_* HF_C_ | -56604.0 | -10886.3 | 0.304 |  |
