# Supplementary material for: Human brain integrates both unconditional and conditional timing statistics to guide expectation and behavior
Source: PLoS Biol. 2025 Oct 23;23(10):e3003459. doi: 10.1371/journal.pbio.3003459 (PMC12561982; doi:10.1371/journal.pbio.3003459)
Supplement: S4 Table — (DOCX) [file pbio.3003459.s005.docx]

| RTs | *After long FP1* | | | *After short FP1* | |
| --- | --- | --- | --- | --- | --- |
|  | *L (LL)* | *S (LS)* | *S (SS)* | | *L(SL)* |
| Block 1 | 0.222$\pm$ 0.033 |  | 0.228$\pm$ 0.029 | |  |
| Block 2 |  | 0.249$\pm$ 0.032 |  | | 0.230$\pm$ 0.038 |
| Block 3 | 0.226$\pm$ 0.036 | 0.259$\pm$ 0.036 | 0.233$\pm$ 0.034 | | 0.228$\pm$ 0.032 |
| Block 4 | 0.233$\pm$ 0.042 | 0.254$\pm$ 0.038 | 0.241$\pm$ 0.029 | | 0.230$\pm$ 0.040 |

Mean $\pm$ Standard deviation. *LL*: RT following long FP2 after long FP1. *LS*: RT following short FP2 after long FP1. *SS*: RT following short FP2 after short FP1. *SL*: RT following long FP2 after short FP1.
