# Supplementary material for: Human brain integrates both unconditional and conditional timing statistics to guide expectation and behavior
Source: PLoS Biol. 2025 Oct 23;23(10):e3003459. doi: 10.1371/journal.pbio.3003459 (PMC12561982; doi:10.1371/journal.pbio.3003459)
Supplement: S5 Table — (DOCX) [file pbio.3003459.s006.docx]

| Block 1 | Block 2 | Block 3 | Block 4 |
| --- | --- | --- | --- |
| 2.12 $\pm$ 1.49 | 1.94 $\pm$ 1.14 | 2.25 $\pm$ 1.16 | 2.69 $\pm$ 1.76 |
| Repeated measures ANOVA; F(3,90) = 2.2425; *p* = 0.088737 | | | |
