# Supplementary material for: Human brain integrates both unconditional and conditional timing statistics to guide expectation and behavior
Source: PLoS Biol. 2025 Oct 23;23(10):e3003459. doi: 10.1371/journal.pbio.3003459 (PMC12561982; doi:10.1371/journal.pbio.3003459)
Supplement: S6 Table — (DOCX) [file pbio.3003459.s007.docx]

| (False alarm trials removed) | Block 1 | Block 2 | Block 3 | Block 4 |
| --- | --- | --- | --- | --- |
| Frist 20 trials | 0.229 $\pm$ 0.028 | 0.236 $\pm$ 0.030 | 0.235 $\pm$ 0.033 | 0.237 $\pm$ 0.034 |
| Last 20 trials | 0.233 $\pm$ 0.030 | 0.242 $\pm$ 0.038 | 0.241 $\pm$ 0.039 | 0.246 $\pm$ 0.043 |
| Pairwise t test | t(30) = -1.3340;  *p* = 0.1922 | t(30) = -0.9546;  *p* = 0.3474 | t(30) = -1.2968;  *p* = 0.2046 | t(30) = -1.6998;  *p* = 0.0995 |
|  | | | | |
| All trials | 0.231 $\pm$ 0.029 | 0.239 $\pm$ 0.031 | 0.237 $\pm$ 0.033 | 0.241 $\pm$ 0.036 |

Mean $\pm$ Standard deviation.
