# Supplementary material for: Human brain integrates both unconditional and conditional timing statistics to guide expectation and behavior
Source: PLoS Biol. 2025 Oct 23;23(10):e3003459. doi: 10.1371/journal.pbio.3003459 (PMC12561982; doi:10.1371/journal.pbio.3003459)
Supplement: S7 Table — (DOCX) [file pbio.3003459.s008.docx]

|  | **Estimates** | **SE** | **β** | ***t value*** | ***p*** | ***Con R^2^*** |
| --- | --- | --- | --- | --- | --- | --- |
| (Intercept) | 5.460 | 0.022 |  | 244.26 | <0.001 | 0.181 |
| HF_U_ | -0.104 | 0.007 | -0.10 | -15.15 | <0.001 |  |

*n* = 17705 observations. Random effect: participants. Estimate: correlation coefficient. β: standardized beta coefficients. SE: standard error. Con R^2^: conditional R-squared.
