# Supplementary material for: Human brain integrates both unconditional and conditional timing statistics to guide expectation and behavior
Source: PLoS Biol. 2025 Oct 23;23(10):e3003459. doi: 10.1371/journal.pbio.3003459 (PMC12561982; doi:10.1371/journal.pbio.3003459)
Supplement: S8 Table — (DOCX) [file pbio.3003459.s009.docx]

| Fixed effects | **AIC** | **BIC** | ***p*** | ***Con R^2^*** |
| --- | --- | --- | --- | --- |
| ~ HF_U_ | 3692.9 | 3731.8 | <0.001 | 0.235 |
| ~ HF_C_ | 3784.0 | 3822.9 | <0.001 | 0.229 |
| ~ HF_U_ + HF_C_ | 3694.7 | 3741.4 | <0.001 | 0.235 |
| ~ HF_U_ + HF_C_ + HF_U_ * HF_C_ | 3596.2 | 3650.7 |  | 0.239 |

*n* = 17793 observations. Random effects: participants and FP1 durations. AIC: Akaike's Information Criterion. BIC: Bayesian Information Criterion. *p* value obtained by comparing the model in the current row to the one in the last row.
