# Supplementary material for: Human brain integrates both unconditional and conditional timing statistics to guide expectation and behavior
Source: PLoS Biol. 2025 Oct 23;23(10):e3003459. doi: 10.1371/journal.pbio.3003459 (PMC12561982; doi:10.1371/journal.pbio.3003459)
Supplement: S9 Table — (DOCX) [file pbio.3003459.s010.docx]

|  | **Estimates** | **SE** | **β** | ***t value*** | ***p*** | ***Con R^2^*** |
| --- | --- | --- | --- | --- | --- | --- |
| (Intercept) | 5.462 | 0.031 |  | 174.53 | <0.001 | 0. 239 |
| HF_U_ | -0.269 | 0.020 | -0.27 | -13.20 | <0.001 |  |
| HF_C_ | -0.036 | 0.009 | -0.04 | -3.90 | <0.001 |  |
| HF_U_ * HF_C_ | 0.225 | 0.022 | 0.24 | 10.04 | <0.001 |  |

*n* = 17793 observations.
