# Supplementary material for: Human brain integrates both unconditional and conditional timing statistics to guide expectation and behavior
Source: PLoS Biol. 2025 Oct 23;23(10):e3003459. doi: 10.1371/journal.pbio.3003459 (PMC12561982; doi:10.1371/journal.pbio.3003459)
Supplement: S10 Table — (DOCX) [file pbio.3003459.s011.docx]

|  | **Estimates** | **SE** | **β** | ***t value*** | ***p*** | ***Con R^2^*** |
| --- | --- | --- | --- | --- | --- | --- |
| (Intercept) | 0.245 | 0.006 |  | 43.02 | <0.001 | 0.173 |
| actual HF_U_ | -0.018 | 0.002 | -0.08 | -11.96 | <0.001 |  |

*n* = 17705 observations.
