# Supplementary material for: Human brain integrates both unconditional and conditional timing statistics to guide expectation and behavior
Source: PLoS Biol. 2025 Oct 23;23(10):e3003459. doi: 10.1371/journal.pbio.3003459 (PMC12561982; doi:10.1371/journal.pbio.3003459)
Supplement: S11 Table — (DOCX) [file pbio.3003459.s012.docx]

| Fixed effects | **AIC** | **BIC** | ***p*** | ***Con R^2^*** |
| --- | --- | --- | --- | --- |
| ~ actual HF_U_ | -48403.9 | -48365.0 | <0.001 | 0.220 |
| ~ actual HF_C_ | -48329.4 | -48290.5 | <0.001 | 0.215 |
| ~ actual HF_U_ + HF_C_ | -48404.5 | -48357.8 | <0.001 | 0.220 |
| ~ actual HF_U_ + HF_C_ + HF_U_* HF_C_ | -48414.2 | -48359.7 |  | 0.221 |

*n* = 17793 observations.
