# Supplementary material for: Human brain integrates both unconditional and conditional timing statistics to guide expectation and behavior
Source: PLoS Biol. 2025 Oct 23;23(10):e3003459. doi: 10.1371/journal.pbio.3003459 (PMC12561982; doi:10.1371/journal.pbio.3003459)
Supplement: S12 Table — (DOCX) [file pbio.3003459.s013.docx]

|  | **Estimates** | **SE** | **β** | ***t value*** | ***p*** | ***Con R^2^*** |
| --- | --- | --- | --- | --- | --- | --- |
| (Intercept) | 0.241 | 0.007 |  | 34.52 | <0.001 | 0. 221 |
| actual HF_U_ | -0.029 | 0.005 | -0.15 | -5.93 | <0.001 |  |
| actual HF_C_ | -0.005 | 0.002 | -0.03 | -3.14 | 0.002 |  |
| actual HF_U_ * HF_C_ | 0.019 | 0.005 | 0.11 | 3.42 | <0.001 |  |

*n* = 17793 observations.
