# Supplementary material for: Human brain integrates both unconditional and conditional timing statistics to guide expectation and behavior
Source: PLoS Biol. 2025 Oct 23;23(10):e3003459. doi: 10.1371/journal.pbio.3003459 (PMC12561982; doi:10.1371/journal.pbio.3003459)
Supplement: S13 Table — (DOCX) [file pbio.3003459.s014.docx]

|  | **Estimates** | **SE** | **β** | ***t value*** | ***p*** | ***Con R^2^*** |
| --- | --- | --- | --- | --- | --- | --- |
| (Intercept) | 0.244 | 0.007 |  | 34.93 | <0.001 | 0. 222 |
| HF_U_ | -0.050 | 0.005 | -0.22 | -10.52 | <0.001 |  |
| HF_C_ | -0.009 | 0.002 | -0.04 | -4.08 | <0.001 |  |
| HF_U_ * HF_C_ | 0.042 | 0.005 | 0.19 | 8.10 | <0.001 |  |

*n* = 17439 observations.
