# Supplementary material for: Human brain integrates both unconditional and conditional timing statistics to guide expectation and behavior
Source: PLoS Biol. 2025 Oct 23;23(10):e3003459. doi: 10.1371/journal.pbio.3003459 (PMC12561982; doi:10.1371/journal.pbio.3003459)
Supplement: Attachment — Submitted filename: reviewers_comments_v5.pdf [file pbio.3003459.s030.pdf]

Reviewer #1: The authors have replied to all of my comments in writing and provided several additional analyses. To facilitate easy reference to the previous round of review, I briefly reply to the authors in a comment-by-comment fashion below. But first, let me preface my review with an important aspect of the modeling work that has not yet been resolved in the revised manuscript.

Preface
The revised manuscript does not offer convincing evidence that the hazard-function-based models provide an adequate fit to the reaction time data over the range of foreperiods.

Quantitatively, the goodness-of-fit is weak (numerically small adj. R2: FP1: 0.173, Table 1; FP2: 0.215 to 0.221, Table 2). Also the beta values are very small (-0.008 to -0.049) indicating weak correlation. Suppl. Fig. 2 depicts this weak correlation.

We thank the reviewer for the careful evaluation and for highlighting important issues regarding the model fit and interpretation of our results. Before addressing specific concerns, we would like to clarify a terminology issue. In the original manuscript, we mistakenly referred to the model fit metric as “adjusted R^2”. However, since we used a linear mixed-effects model, the appropriate metric is “conditional R^2”, which accounts for both fixed and random effects. Importantly, this was a labeling error only; the calculations and reported values are correct and remain unchanged. We have revised the terminology throughout the manuscript and supplementary materials to accurately reflect this correction. Below, we respond point by point to the comments.

Response 1: Model fit
We acknowledge the reviewer’s concern regarding the modest conditional R^2 values (FP1: 0.173; FP2: 0.215–0.221). These values were obtained from analyses in which only false alarm trials were excluded, while outliers and the first 20 trials of each block were retained. This decision was made in response to the previous *Comment 4* and reflects our approach to minimize assumptions about the underlying processes of outlier trials.

To examine whether trial-level noise from outliers contributed to the modest fit, we conducted a follow-up analysis using a stricter dataset in which both false alarms and RT outliers (defined as ±2.5 SD) were excluded. This resulted in consistently improved conditional R^2 values (see details below):

- **FP1**: Conditional R^2 increased to 0.262
- **FP2**: Conditional R^2 increased to 0.295–0.304

These updated results are now reported in **Supplementary Table 3**. The main text has also been revised to indicate that trial-level variability attenuated the explanation of the relationship between HF and RT in our original analysis. Additionally, we tested model fit using log-transformed RTs and obtained conditional R^2 values of 0.301 for FP1 and 0.340–0.353 for FP2.

However, in the main text, we chose to retain the original data selection and analysis approach, which excludes only false alarms and uses non-transformed RTs. This decision was based on two

considerations: first, to avoid introducing additional assumptions about the underlying processes of outliers and the assumption of normally distributed reaction times; and second, although the R^2 values are not high, they are reasonable compared to those reported in existing publications, as we will discuss below.

Supplementary Table 3. Comparisons of linear mixed-effects models on reaction times (*false alarms only vs. false alarms and reaction times outliers excluded*)

Fixed effects	AIC	BIC	*Adj R^2*
RT following FP1 (false alarm removed)			
~ HF_U	-43719.0	-43687.8	0.173
RT following FP1 (false alarm & outliers removed)			
~ HF_U	-54015.2	-53984.2	0.262
RT following FP2 (false alarm removed)			
~ HF_U	-48388.5	-48349.6	0.218
~ HF_C	-48335.4	-48296.5	0.215
~ $HF_U + HF_C$	-48386.8	-48340.1	0.218
~ $HF_U + HF_C + HF_U{*} HF_C$	-48449.7	-48395.1	0.221
RT following FP2 (false alarm & outliers removed)			
~ HF_U	-56512.3	-10776.3	0.300
~ HF_C	-56423.5	-10630.9	0.295
~ $HF_U + HF_C$	-56510.5	-10767.9	0.300
~ $HF_U + HF_C + HF_U{*} HF_C$	-56604.0	-10886.3	0.304

Comparison with previous studies

Our study yielded modest conditional R^2 values, but they are reasonable when compared to prior studies, particularly considering: (1) the use of a more complex probability distribution structure, (2) the inclusion of a full range of foreperiods, and (3) the introduction of both unconditional and conditional probability regularities within a single task. We elaborate on each of these below and summarize them in the table that follows.

Representative Study	Probability Distribution	Trial Inclusion	Task complexity	R^2
**Herbst et al. (2018)**	Unimodal, Uniform	Full FP range used	Unconditional probability	Con. R^2<br>0.429, 0.448
**Grabenhorst et al. (2019)**	Exponential, Flipped	Full FP range used	Unconditional probability	Adj. $R^2 \approx 1$ (Supplementary Figure 13)
**Janssen & Shadlen (2005)**	Bimodal, Unimodal	Trials between peaks excluded	Unconditional probability	Adj. R^2 =<br>0.95, 0.96
**Bueti & Macaluso (2010)**	Bimodal	Trials between peaks excluded	Unconditional probability	$R^2 = 0.53$
**Our Study**	Bimodal for FP1<br>Biomodal and Skewed Unimodal [only Blocks 1 and 2] for FP2	Full range used (except 1.2 s FP)	Unconditional & conditional probabilities	Con. R^2 = 0.17–0.20 (main), 0.26–0.30 (outliers removed), 0.30–0.35 (log-transformed)

				* PDF and all blurring effects were tested and the results were similar

1. Studies using simple probability distributions yield better fit

For instance, Herbst et al. (2018) applied a linear mixed-effects model using log-transformed RTs and HF as a fixed effect, reporting conditional R^2 values of 0.429–0.448 for unimodal and uniform distributions, respectively. On the other hand, Grabenhorst et al. (2019) reported even higher model fits (adjusted R^2 values approaching 1; see their Supplementary Figure 13) using exponential and flipped distributions without catch trials.

Although these studies reported higher R^2 values, a key difference lies in the simplicity of their probability structures, which typically produce hazard functions or probability distributions with a single peak or monotonic increase used for model fitting. In contrast, our bimodal distributions generate dual expectancy peaks, resulting in more complex temporal expectations that cannot be adequately captured, such as, by a simple monotonic drift in brain signals.

2. Design issues in studies using bimodal probability distributions

Some studies have reported high R^2 values with bimodal distributions. For example, Janssen and Shadlen (2005) reported values of 0.95 and 0.96 in two non-human primates, and Bueti and Macaluso (2010) reported a value of 0.53 in humans. However, these studies did not include a full range of foreperiods. In Janssen and Shadlen (2005), for instance, the bimodal distribution had peaks around 0.27 and 1.93 seconds, but no trials were presented between 0.75 and 1.75 seconds (i.e., between the peaks). There is the omission of intermediate foreperiods with low probability, where presumably few trials are set and high uncertainty could be caused. This design reduces trial-by-trial variability and likely improves model fit by this omission.

In contrast, our design incorporated a nearly continuous range of foreperiods (excluding only 1.2 seconds to avoid categorical confusion between short and long FPs). This allowed us to capture the full dynamics of the hazard function, providing a more comprehensive and naturalistic test of temporal prediction over time. However, this approach also introduced greater variability, which can reduce R^2. Notably, this comprehensive design was not limited to our study but also adopted in the above studies using a simpler probability structure.

3. Task complexity

Finally and most critically, our study is the first to combine two probability distributions within a single task. Moreover, while employing a bimodal distribution, we also varied its structure across blocks (e.g., equal 50–50 peak contributions vs. asymmetric 20–80 peaks). This manipulation was essential for disentangling unconditional and conditional probability structures and also for assessing how the brain generalizes across different probabilistic rules.

Such complexity can increase trial-by-trial (and also inter-individual variability) in learning and strategy, which in turn can reduce the explained variance in model fitting. Still, we would like to emphasize that despite this added complexity, our key behavioral and neural findings remain robust and statistically significant. All major results, including significant behavioral and neural correlations with HF_U, HF_C, and the interaction term, were consistently verified and shown through control and additional analyses.

We have included the Supplementary Table 3 in the revised Methods:

> "While trial-level variability from outliers attenuated the model fit (see the comparisons in Supplementary Table 3), we emphasize that the overall conclusions remain unchanged, and the robustness of our results can be further demonstrated by analyses using nearly the entire dataset." (Page 30, Lines 15-18)

We have also discussed this in the revised Discussion:

> "We also acknowledge that the conditional R^2 values in our behavioral data appear modest, and the relatively poor model fits can be attributed to several factors. First, we included reaction time outliers and used non-log-transformed data in our analysis (see details in Methods) to avoid introducing additional assumptions. We verified the contribution of trial-level noise from outliers to the model fit by demonstrating consistently improved conditional R^2 values when outliers were removed (Supplementary Table 3). Second, unlike previous studies that used exponential or unimodal probability distributions, typically resulting in a single peak or a monotonic increase, we employed bimodal distributions that generate dual expectancy peaks. This probability structure produces more complex temporal expectations that cannot be adequately captured by a simple monotonic drift in brain signals. Third, to ensure a complete probability distribution structure, we included trials across the full foreperiod range in both our design and analysis, even though some foreperiod durations were in few trial numbers, likely introducing trial noise. This allowed us to more comprehensively capture the evolution of the hazard function over time. Finally, our design is the first to create two probability regularities, and to achieve it, we also designed various bimodal distributions across blocks (e.g., equal 50-50 peak contributions vs. asymmetric 20-80 peaks). Such complexity might increase trial-by-trial (also inter-individual) variability in learning and strategy, which in turn can increase behavioral noise and reduce the explained variance in models. Still, we would like to emphasize that despite this added complexity, our key behavioral and neural findings remain robust. All major results, such as both significant behavioral and neural correlates with HF_U, HF_C, and the interaction term, were consistently verified and shown through control and additional analyses." (Pages 21-22 Lines 16-13)

Response 2: Beta coefficients

The reported beta values were unstandardized and therefore influenced by the scale of the predictor variables. To improve interpretability, we have added standardized beta coefficients, which range from 0.04 to 0.21 across models. While modest in magnitude, these effects are statistically significant and consistent across models and analyses. The standardized beta values have been incorporated into **Tables 1 and 3** (also shown below) as well as **Supplementary Tables 7, 9, 10, and 12**.

Table 1. Effect of HF_U on reaction times following FP1

	Estimates	SE	β	*t value*	*p*	*Con R^2*
(Intercept)	0.244	0.006		42.88	<0.001	0.173
HF_U	-0.021	0.002	-0.08	-11.67	<0.001	

n = 17705 observations. Random effect: participants. Estimate: correlation coefficient. β: standardized beta coefficients. SE: standard error. Con R^2: conditional R-squared.

Table 3. Effects of HF_U and HF_C on reaction times following FP2

	Estimates	SE	β	*t value*	*p*	*Con R^2*
(Intercept)	0.243	0.007		34.93	<0.001	0. 221
HF_U	-0.049	0.005	-0.21	-10.44	<0.001	
HF_C	-0.008	0.002	-0.04	-3.93	<0.001	
HF_U * HF_C	0.042	0.005	0.19	8.06	<0.001	

n = 17793 observations.

Qualitatively, the RT modulation (Suppl. Fig. 1) does not resemble the dynamics of the (mirrored) HF variables (Fig 1E).

Since all of the manuscript's main results hinge on the HF fits to RT, more must be done to convince the reader that the HF is indeed an appropriate model of RT (and a neurobiologically relevant computation).

First, it is crucial for the reader to see the average RT over foreperiod (group-level) plots in the manuscript (not in the Supplement) so that the reader can assess the modulation of RT.

Second, these average RT over foreperiod (group-level) plots need to show the dynamics of the RT modulation in a clear way. The current Suppl. Fig. 1 does not do this sufficiently: The y-axis spans close to 1 s, while the RT modulation spans less than 0.1 s, making it hard to see any pattern. All the boxes, error bars, outlier dots etc. in the plots further obscure a clear view of the RT modulation.

The authors need to plot average RT over foreperiod and rescale the y-axis so that the RT modulation over foreperiods becomes clearly visible. See e.g. these papers for intuitive average RT over foreperiod plots: Janssen & Shadlen, Nat Neuro 2005, Bueti et. al. J Neurosci 2010, Grabenhorst et al. Nat Comms 2019, Schoffelen et al., Science 2005.

*Third, Suppl. Fig. 2 shows RT over hazard. This figure does not show the fits *over time*, i.e. over foreperiods. Instead, in Suppl. Fig. 2, data are collapsed across foreperiods. It is therefore not possible to visually inspect the model fits to data *over time* (i.e. over foreperiod). Such an assessment is crucial since a convincing model needs to capture RT dynamics over the range of foreperiods. The average RT over foreperiod plots are the common way to display fits to data in these types of experiments (e.g. Fig. 2 in Janssen & Shadlen, Nat Neuro 2007, which the authors cite).*
*The authors need to provide plots that show how the HR model fits *group-level* average RT *over time*, i.e. over foreperiods. This should be done for *all models* so that the reader can assess the choice of models based on visual inspection and not only based on fit metrics (adj. R2, AIC, BIC).*
[n.b. The authors say that it is challenging to visualize the interaction term in one of their models (their resply to comment 5). Some ideas: 3D surface plots, line plots across time for different levels of HFX(t), contour plots...)

Response 3: Visualizing average RT over foreperiods and model fit across all models
We fully agree with the reviewer that the original Supplementary Figure 1 did not clearly demonstrate RT changes due to its wide y-axis range and overly detailed plotting elements (e.g., boxplots and outliers). In response, we have created **a new Figure 2** that shows group-level average RTs over foreperiods in FP1 and FP2 across all four blocks, using a more appropriate y-axis range and simplified plotting (mean with standard deviation shading only). This figure also includes: (1) the predicted RT values following FP1 from Model 1 (HF_U only), (2) the predicted values following FP2 from Model 4 (HF_U, HF_C, and the interaction term), and (3) the actual trial distribution across all foreperiods at the bottom.

We elaborate further on them in Response 4 below. We sincerely thank the reviewer for this suggestion, which helped us overcome the challenge of visualizing Model 4 (HF_U, HF_C, and the interaction term). Accordingly, we have also revised the visualization of model fits from other models in **Supplementary Figure 1** and used actual HF values in **Supplementary Figure 14**.

Figure 2

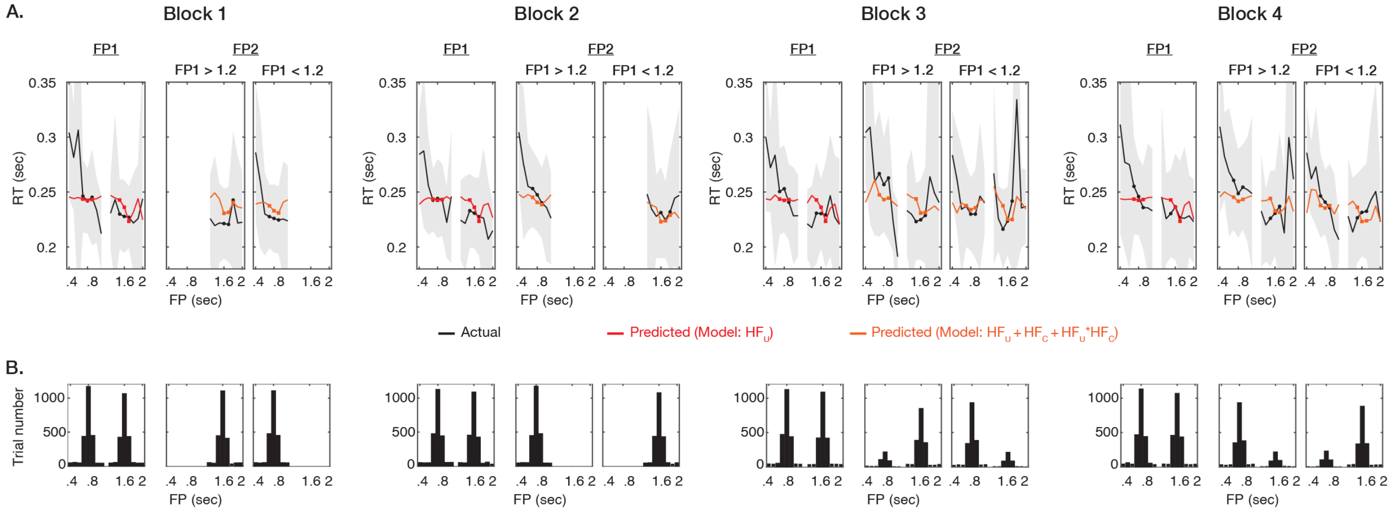

Supplementary Figure 1

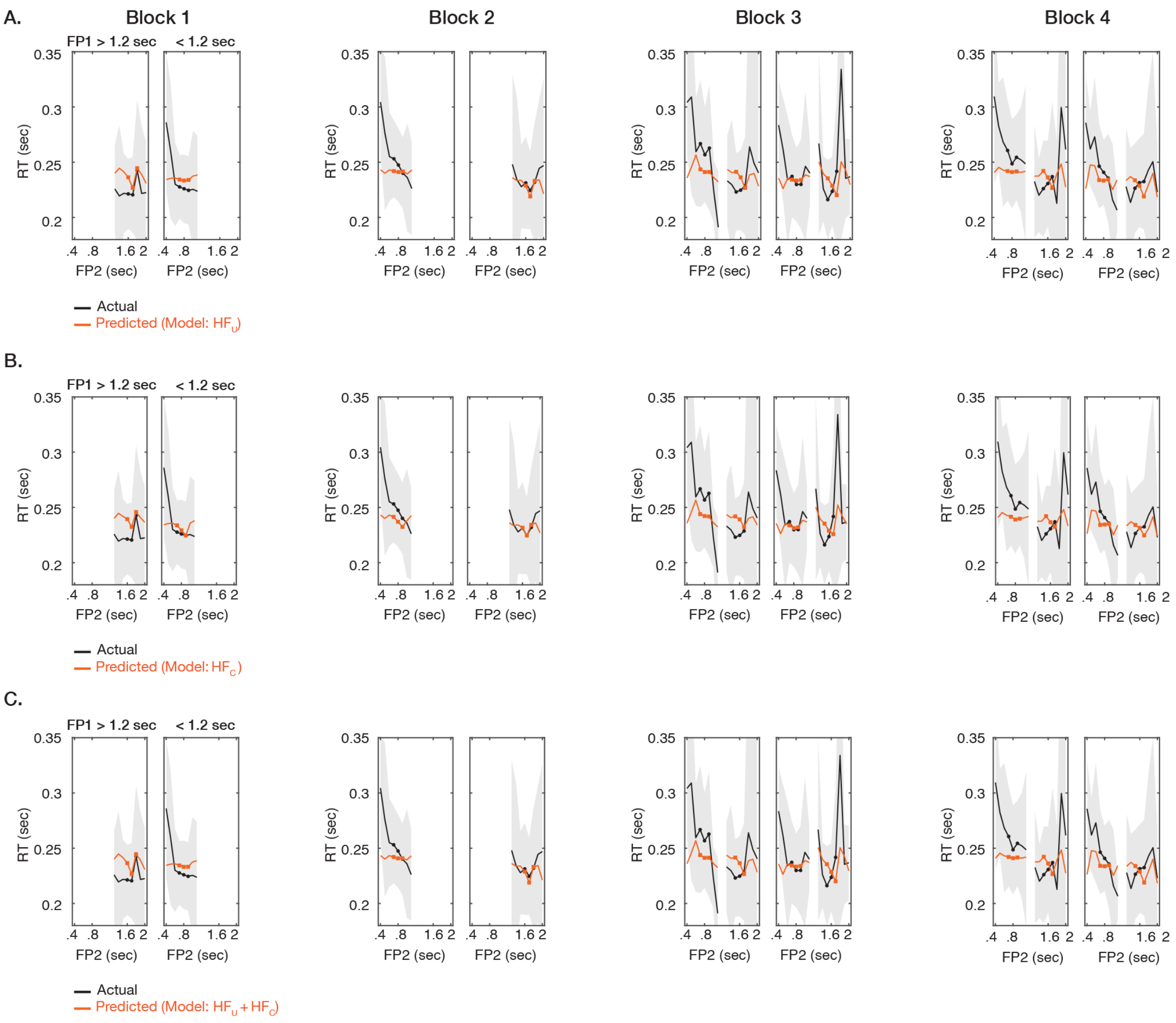

Response 4: Model fit interpretations

We agree with the reviewer that both visual inspection and statistical criteria (e.g., AIC, BIC) are critical for evaluating model performance. Several key points should be emphasized regarding the interpretation of model fits in the new Figure 2.

First and also before discussing the model fit in detail, it is important to note that we performed the linear mixed-effects model analysis using data from all four blocks combined. Accordingly, the predicted and actual RTs across all blocks should be considered jointly when evaluating model fit. For example, inspecting the model fit for FP2 involves considering the eight actual and predicted RT curves across the various blocks together.

Second, we note that the predicted RT values (red and orange) generally fall within the range of the observed group mean (black) ± 1 standard deviation (shading), indicating an overall reasonable fit. However, some discrepancies in the trend can be observed, particularly at short foreperiods (e.g., 0.4–0.6 s), where the actual RTs show a drop (black), while the predicted RTs remain relatively flat (red and orange). Several points are worth noting in this context:

1. The predicted values in these early foreperiods are flat because they were associated with very low probability values, deliberately designed to approach zero. As a result, the derived HF values (both blurred and unblurred) were also close to zero, producing flat predicted RTs.
2. As discussed in **Response 1**, although these short foreperiod trials were few in number, they were retained to preserve the full hazard function change over time. This approach naturally led to increased variability, reflected in the wider standard deviation shading in those areas.
3. A similar pattern, where RTs vary while HF remains stable, has also been observed in previous work using longer-tailed probability distributions with sparse trials at both ends, such as Herbst et al. (2018).

To aid visual interpretation, we marked the foreperiods with dominant trial frequencies (0.7, 0.8, 0.9, 1.5, 1.6, and 1.7 s) using dots. These highlighted points more clearly demonstrate that the observed RTs correspond well with both the predicted trend and the peaks in the foreperiod distribution.

Finally, although the model fits for FP2 in **Figure 2** and **Supplementary Figure 1** appear visually comparable across the four models, statistical comparisons using AIC and BIC nonetheless identified Model 4 (which includes the interaction term) as the best-fitting model.

Accordingly, we have revised the Results section.

> "We also visualized the observed and predicted reaction times across all foreperiods (Figure 2A). A general trend consistent with the HF can be observed, particularly in the foreperiods with dominant trial frequencies, where actual and predicted reaction times closely align. Note that data from all blocks were combined for the model analysis; however, we separated them in the figure for clearer illustration of the actual reaction times across foreperiods alongside the predicted values." (Page 9, Lines 4-10)
>
> "We first visualized the actual and predicted reaction times for all models in Figure 2 and Supplementary Figure 1. Due to the varied block-wise probability distributions, visually inspecting model fit is more challenging, as it requires jointly evaluating all predicted curves across the four blocks. To statistically determine the best-fitting model, we performed model comparisons." (Page 10, Lines 2-6)

Comment 1

The authors improved the terminology in their manuscript. This is very helpful, as is the table showing the changes. One minor point: In the intro (P3, l14) the authors say that the hazard rate itself is a conditional probability. While this makes sense in itself, it may throw off the reader who may confuse it with the use of the terms (un-)conditional, as in e.g. Fig. 1D. Maybe rephrase the sentence in the intro to not use the word conditional?

We thank the reviewer for pointing this out. To avoid potential confusion with the terminology used later in the manuscript, we have revised the sentence as follows: "This function, derived

from the probability distribution of the foreperiod, describes how the probability of the target signal occurring is updated over time, given that it has not yet occurred.” (Page 3, Line 13-15)

The authors performed new model fits to non-log-transformed RT. This is in line with the bulk of the hazard rate literature and does not add neuro-computational assumptions as the log-transform did. Both improves interpretability. The authors kept the fits to log-transformed RT and added them to Supplement.

We appreciate the reviewer’s understanding on this point.

The authors reply to my comment that they regressed HF on EEG data, not RT. I am aware of this fact. The crucial point is: if the HF fits to RT are not convincing, this does call into question the HF as an appropriate regressor and thus questions the EEG analysis and the interpretation of the results, see Preface.

We thank the reviewer for pointing this out. We have addressed this comment by providing an improved visualization of the HF across all foreperiods along with the predicted values in **Figure 2** and **Supplementary Figure 1** (see **Responses 3 and 4**). We also show significant and moderate correlations between RT and HF (**Response 2**), and discuss prior literature that supports the use of HF as a regressor (**Response 5** below).

Comment 2
Please see Preface above.

We have addressed this comment by providing **Figure 2** and **Supplementary Figure 1** (see **Responses 3 and 4**).

Comment 3
The authors convincingly addressed this comment.

Comment 4
The authors convincingly addressed this comment.

Comment 5
i) On the model selection: Please see Preface.

We have addressed the comment above in **Responses 3 and 4**.

ii) On the temporal uncertainty:
The authors tested temporally and probabilistically blurred HF models and find the results inconclusive.

Based on the authors' reply to my comment, I get the impression that they think that the different blurring regimes are just a means to improve model fit ("The winner in RT following FP1 is using probabilistically blurred HF. However, the winner in RT following FP2 is using

temporally blurred and probabilistically blurred HF." and "However, the optimal blurring effect varies, depending on different blurring factors").

Instead, these blurring regimes serve to test specific hypotheses about the brain's uncertainty in time estimation.

*It is widely accepted that there are errors to neural temporal estimates, e.g. in interval timing. These timing mechanisms are crucial for sucessful performance of the timing task that the authors report here. Therefore, the reader could expect the authors to take a stance on uncertainty in time estimation, especially since the manuscript's abstract closes with this bold statement: "Our study reveals brain networks that integrate multilevel temporal information, offering insight into the hierarchical predictive coding of time." It is worth noting that the manuscript's modeling work unrealistically implies that time is encoded *perfectly* in the brain, without any error....*

The authors state in the Discussion (p 18, l. 18-20): "Therefore, although we believe that the time estimation during waiting is unlikely to be equally precise throughout, we chose to use a simpler model that does not include explicit blurring parameters or assumptions about precision."

To the contrary, the non-blurred models that the authors identified as the best-fitting ones make a specific assumption about precision, i.e. that the brains studied in this experiment are perfect clocks that make no timing errors (and can perfectly estimate two hazard rates simultaneously). This seems to be a neurobiologically implausible assumption, given the literature on time estimation (Gibbon Psychol Rev 1977, Gallistel & Gibbon Psychol Rev 2000).

Response 5: On using different blurring factors and parameters (also dddressing the third question below)
We sincerely appreciate the reviewer’s comments regarding the role of uncertainty in time estimation. We acknowledge that our previous response may have unintentionally given the impression that temporal and probabilistic blurring were used solely to improve model fit. We would like to clarify that our intention in testing these different blurring factors was to evaluate competing hypotheses about the neural representation of uncertainty during time processing.

To our knowledge, the question of how the brain represents uncertainty in temporal prediction remains unresolved in the existing literature. For example, neural activity in the primate brain has been shown to closely correlate with temporally blurred HFs under both unimodal and bimodal distributions (Janssen & Shadlen, 2005). In long-foreperiod paradigms, where greater temporal uncertainty is expected, RTs have also been successfully explained by temporally blurred HFs (Bueti et al., 2010; Bueti & Macaluso, 2010). Conversely, other studies suggest that probabilistically blurred probability distribution itself (PDF) better account for human behavioral data (Grabenhorst et al., 2019, 2021). Still, unblurred HFs have also been effectively used to differentiate behavioral and neural responses across uniform and unimodal distributions (Herbst et al., 2018), in both explicit and implicit timing tasks (Coull & Nobre, 2008), and in modeling trial-by-trial neural responses to surprise in the foreperiod paradigm (Visalli et al., 2021).

These seemingly conflicting findings may reflect important differences across studies (Nobre & van Ede, 2018), including: (1) the statistical properties of the foreperiod distributions (e.g., mean and variance); (2) the temporal resolution of the experimental design; and (3) the use of catch trials. These factors can result in varied temporal prediction profiles, along with different types and magnitudes of uncertainty. For example, regarding the first point, in a bimodal distribution formed by overlapping two identical unimodal distributions, increasing the distance between the peaks can amplify temporal blurring. This is evident in the ~0.27- and ~1.93-second peaks in Janssen & Shadlen (2005) and the ~3.91- and ~13.75-second peaks in Bueti et al. (2010), compared to the 0.8- and 1.6-second peaks in the current study. For probabilistic blurring, the degree of blurring can be influenced by the standard deviation of the distribution, a narrower distribution produces sharper changes over time, whereas a flatter distribution leads to more gradual changes.

Regarding the second point, even when foreperiod distributions have identical means and standard deviations, the overall range can still affect uncertainty. For instance, a longer range (0–20 seconds, as in Bueti et al., 2010) can introduce greater temporal uncertainty than a shorter range (0.4–2 seconds), as used in our study. Furthermore, it is also reasonable to infer that both temporal and probabilistic blurring may coexist and interact with each other when using probability distributions with different means, standard deviations, and time ranges. In a side note, temporal resolutionm, defined by the time step used in the analysis (e.g., 0.1-second vs. 0.01-second), can also influence the dynamics of the HF, particularly around the later peaks. This effect is illustrated in our simulations based on Bueti et al. (2010) and our own study, as well as in comparisons with Herbst et al. (2018) (see green arrows in the figures below).

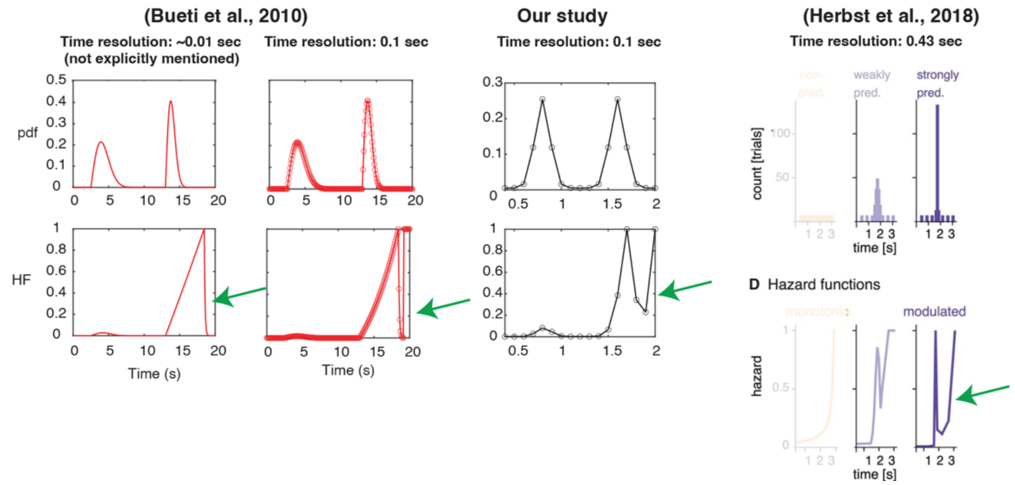

Third, the inclusion of catch trials in some foreperiod paradigms not only introduces additional uncertainty regarding whether the target will occur but also complicates the definition of the probability distribution for target trials. Regarding the former, Grabenhorst et al. (2021) modeled RT by incorporating both the uncertainty of target occurrence and probabilistic uncertainty (i.e., probabilistic blurring), raising important questions about how these two forms of uncertainty interact in the brain. Regarding the latter, distribution estimation becomes more complex, as catch trials may either be omitted or interpreted as extremely long foreperiods within the distribution.

Returning to our study, which aimed to test whether the brain can learn and integrate both unconditional and conditional temporal statistics, we implemented distinct foreperiod distributions that inevitably introduced different types of uncertainty across conditions. We found that no single blurring factor consistently outperformed others across FP1 and FP2, further supporting our first and second points. Importantly, we do not claim that the original HF consistently yielded the best-fitting model. Rather, we emphasize that the integration of multilevel temporal information was consistently observed in any blurring factors used. Notably, all better-fitting models were based on hazard functions—either temporally or probabilistically blurred. Consistent with prior neurophysiological findings demonstrating neural encoding of original hazard functions, our results revealed significant associations between RT/neural activity and original HF predictors. This suggests that the brain may perform HF-like computations as a mechanism for temporal prediction, with different forms of uncertainty modulating these estimates in a context-dependent and potentially parallel manner.

Once again, we fully acknowledge the reviewer's important point: non-blurred models imply a simplified assumption that time is encoded without error. While this assumption allows us to isolate core computational principles, it underrepresents the role of uncertainty. Its modulatory effects should be further investigated in future research, such as using foreperiod ranges of different lengths (to test temporal blurring) or manipulating the standard deviation of distributions (to test probabilistic blurring). Accordingly, we have explicitly discussed this limitation in the revised Discussion to better reflect the complexity of the underlying temporal processes.

Discussion section:

> "Still, in long-foreperiod paradigms, where greater temporal uncertainty would be expected, RTs have been well explained by temporally blurred HFs (Bueti et al., 2010). These seemingly conflicting findings across studies may reflect key differences across studies (Nobre & van Ede, 2018), including: (1) the statistical properties of the foreperiod distributions (e.g., mean and standard deviation), (2) the temporal resolution of the experimental design, and (3) the use of catch trials. These factors can introduce varied temporal prediction profiles with different types and magnitudes of uncertainty. Regarding the first point, in a bimodal distribution created by overlapping two identical unimodal distributions, a greater distance between the peaks is expected to produce a stronger temporal blurring effect. For probabilistic blurring, the extent of the effect can be influenced by the distribution's standard deviation, for instance, a sharper distribution may lead to more rapid changes over time than a flatter one. For the second point, even when distributions share the same mean and standard deviation, the range of foreperiods can still affect uncertainty. For example, a longer range (e.g., 0–20 seconds, as in Bueti et al., 2010) introduces greater temporal uncertainty than a shorter range (e.g., 0.4–2 seconds, as used in our study). It is also reasonable to infer that temporal and probabilistic blurring may coexist and interact, potentially modulating one another, when using probability distributions with different means, standard deviations, and time ranges. Third, the inclusion of catch trials not only adds uncertainty regarding whether the target will occur but also complicates the definition of the underlying probability distribution. For instance, Grabenhorst et al. (2021) modeled RT by incorporating both the uncertainty of target occurrence and probabilistic uncertainty (i.e., probabilistic blurring), raising the

> question of how these two forms of uncertainty interact in the brain. In terms of probability distributions, catch trials may either be excluded or interpreted as extremely long foreperiods, making distribution estimation more complex.
> …
> This inconsistency further supports our earlier points: in our study, we implemented distinct foreperiod distributions to examine whether the brain can learn and integrate both unconditional and conditional temporal statistics. This design inevitably introduced different types of uncertainty across conditions. As shown in previous neurophysiological work, which demonstrates neural encoding of original hazard functions, and consistent with our findings, we suggest that the brain may rely on HF-like computations as a neural mechanism for temporal prediction. These computations may be modulated by different forms of uncertainty in a context-dependent and potentially parallel manner. Nevertheless, we emphasize that while using non-blurred models allows us to isolate core computational principles, under the simplifying assumption that time is encoded without error, it also underrepresents the role of uncertainty. The modulatory effects of uncertainty should be explored in future research, for example by varying foreperiod ranges (to examine temporal blurring) or manipulating the standard deviation of the distribution (to examine probabilistic blurring)." (Pages 18-20, Lines 18-14)

In fact, it should worry the authors that all versions of their models (non-blurred, temporally blurred, probabilistically blurred, Suppl. Fig. 8) yield rather small goddness-of-fit values (adj. R2), as the authors acknowledge. The authors speculate that this may be due to the number of trials per participant which they say may be too small. Three questions:
1) If this is the case, then how can the main results of the manuscript be conclusive?

Response 6: Trial Numbers
We appreciate the reviewer's concern. When designing our experiment, we determined the number of trials, 600 for FP1 and 600 for FP2, based primarily on prior studies. Specifically, we referred to: (1) Herbst et al. (2018), in which each participant completed a total of 546 trials during EEG recordings; and (2) Bueti et al. (2010), which included 600 trials in a training session and 200 trials in a testing session during fMRI acquisition. Notably, they reported no substantial differences between the results from the two sessions. Based on these references, we specified the rationale for our chosen trial numbers in the Methods section.

In addition, we acknowledge that our earlier attribution of low conditional R^2 values to limited trial numbers per participant was speculative and not supported by direct evidence. To evaluate this more rigorously, we conducted a bootstrapping analysis to assess the stability of R^2 values within our dataset. Specifically, for each participant and for both FP1 and FP2, we resampled their trials with replacement and re-estimated the R^2 value 1,000 times to generate a bootstrap distribution and corresponding 95% confidence intervals. This allowed us to assess whether the observed R^2 values were statistically stable and representative. Please see the results below and in **Supplementary Figures 11 and 12**. Our assessment is based on two criteria: (1) The bootstrap distribution of R^2 should be unimodal. Bimodal or uniform distributions would suggest instability. While skewness toward zero may reflect limited informativeness, it does not necessarily imply instability. (2) The actual R^2 value should fall within the 95% confidence interval of the bootstrap distribution and ideally be close to the bootstrap mean. Across

participants, these criteria were generally met for both FP1 and FP2, suggesting that the number of trials per participant was sufficient to yield statistically stable R^2 estimates. We believe these findings reinforce the robustness of our conclusions.

Methods section:

> "Each participant completed a total of 600 FP1 trials and 600 FP2 trials, with the number of trials determined primarily based on prior studies (Bueti et al., 2010; Herbst et al., 2018). We also confirmed the stability of individual results using bootstrap resampling, which showed that the distribution of individual R-squared values was unimodal, with the actual values close to the bootstrap mean and within the 95% confidence intervals (Supplementary Figures 11 and 12)." (Page 28, Lines 6-11)

Supplementary Figure 11

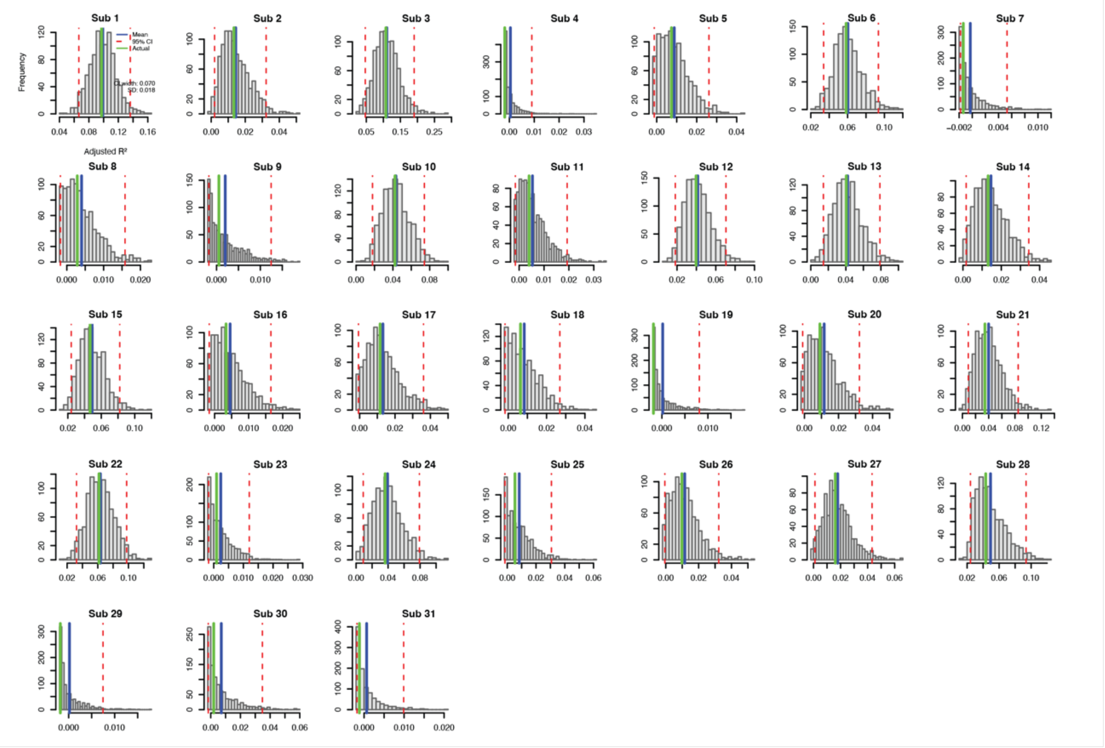

Supplementary Figure 12

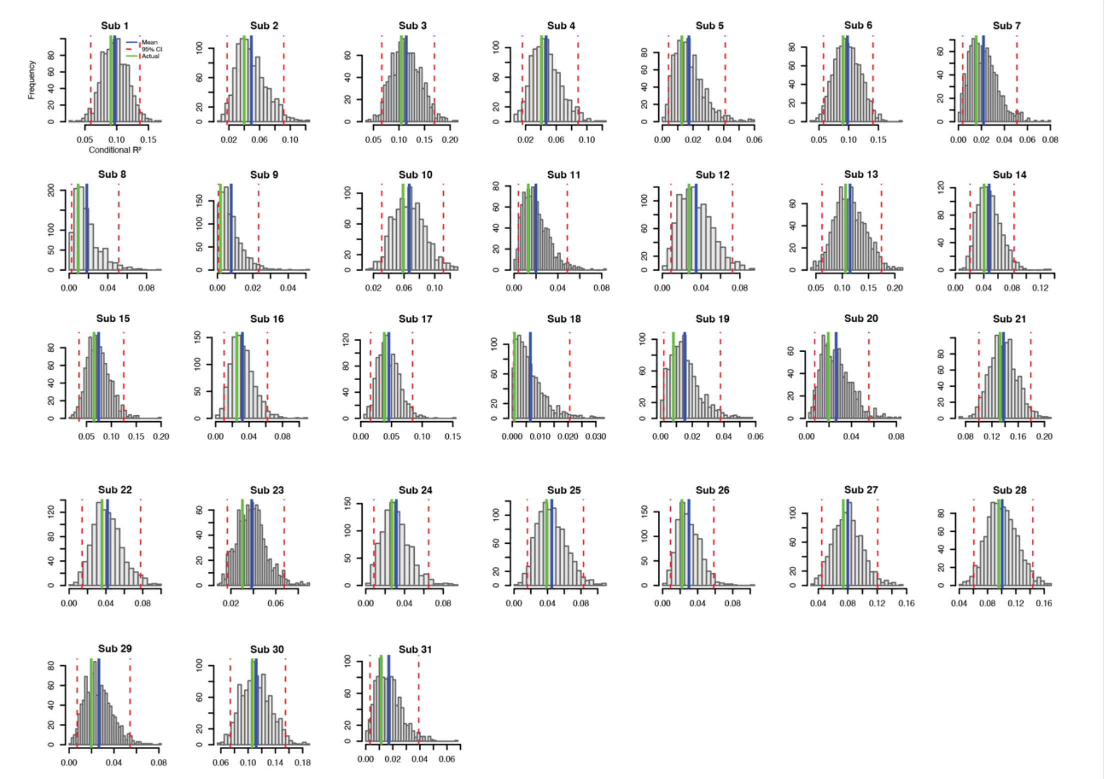

2) Do the HF models fit the group-level RT well (see Preface)?
3) Is the hazard function an adequate model for these data at all?

These two questions have been addressed in Responses 1–5.

Comment 6
The authors convincingly addressed this comment.

Comment 7
*In the Discussion (p. 20 l. 2-3), the authors write: "Here, it is important to clarify again that the integration of two probabilities was supported by the optimal model performance with the inclusion of the interaction term (i.e., HFU * HFC)."*
The term "optimal" is inappropriate here. The authors probably want to express that the model with the interaction term yielded the best fit relative to the other models that did not feature such a term. So this is a relative statement and should be formulated as such. The current sentence implies that the model is performing optimally, which does not make sense.

We thank the reviewer for pointing this out. Accordingly, we have revised the sentence to: "Here, it is important to clarify again that the integration of two probabilities was supported by the model including the interaction term (i.e., HF_U * HF_C), which yielded the best fit relative to the other models that did not feature such a term." (Page 18, Line 5-12)

Comment 8
The authors convincingly addressed this comment.

Comment 9
The authors convincingly addressed this comment.

Comment 10
The authors convincingly addressed this comment.

Reviewer #2 (Samuel A. Nastase): The authors have largely addressed my specific questions. The authors have made substantial improvements to the manuscript, and their results seem fairly robust to analytic choices highlighted by other reviewers. I still have reservations about the generalizability of an experimental manipulation of this kind, but I'm happy to concede the point. I have a couple remaining concerns that were not fully addressed in the revision.

We thank the reviewer for the careful evaluation and for pointing out the need to describe the analyses in more detail.

Regarding the ridge coefficient of equation 10 in the mTRF section of the Methods, we still need some further details as to what's going on here. For example, initially the authors say the ridge parameter was "set to 1", then say the ridge parameter was "based on optimal correlation coefficients between hazard values and EEG signals in the cross validation." These seem like conflicting statements to my reading, and are not written with the mathematical precision of the rest of the manuscript. I assume this is because the mTRF software is doing most of the work internally, but the description still needs to be clearer. If you're setting the ridge parameter to 1 (presumably the default parameter), then you aren't doing any hyperparameter optimization (not optimal).

If you're doing hyperparameter optimization, then each outer cross-validation fold is going to have a different ridge parameter (optimal). To understand what's happening here, we need to understand the cross-validation structure much more clearly.

*First, what exactly is the structure of the *outer* cross-validation loop?*

*Second, if you're optimizing the ridge hyperparameter lambda, the optimal ridge parameter for a given outer fold should be determined using an *inner* cross-validation loop within the training set of that outer fold. Typically for a given fold of the outer loop, you evaluate a grid of different hyperparameter settings (what grid? e.g. lambda values log spaced from the interval X to Y) using nested cross-validation within the training set; then use the best-performing hyperparameter to refit the model on the full (outer) training set and evaluate it on outer test set; then repeat this procedure for each fold of the outer loop. This is completely standard in widely used software, e.g., RidgeCV in scikit-learn. It's possible that this is already what the mTRF software is doing internally, but even so, the authors need to describe it more accurately.*

In our analysis, we used leave-one-out cross-validation as implemented in the *mTRFcrossval* function from the mTRF Toolbox. As the reviewer correctly described, the outer loop treats each trial as one fold. Within each outer fold, the model is trained separately using a set of candidate lambda values ranging from 10^{-3} to 10^{3} in log-space (i.e., 10.^(-3:1:3)) and four candidate time lags, with minimum lags set to either –0.2 or –0.1 seconds and maximum lags to either 0.2 or 0.3 seconds.

Specifically, for each outer fold, one trial is held out as the test set, and the remaining N–1 trials are used for training. For each candidate lambda and time lag, a model is trained on the training set and then evaluated on the held-out trial. This process is repeated for all lambda values and time lags (inner loop) and all trials (outer loop), resulting in performance metrics (i.e., correlation

coefficients) for each lambda value and time lag across all folds. To determine the optimal lambda and time lag, we followed the procedure used in the mTRF Toolbox tutorial and selected the value that yielded the best average performance across outer folds. Again, we thank the reviewer for bringing this up, and we have revised the manuscript accordingly.

Methods section:

> "Specifically, we tested the ridge parameter λ across a log-spaced range from 10^{-3} to 10^{3} (i.e., 10.^(-3:1:3)) and evaluated four combinations of time lag parameters (τ), with minimum lags set to either –0.2 or –0.1 seconds and maximum lags to either 0.2 or 0.3 seconds. In each fold of the outer loop, one trial was held out as the test set, while the remaining *N–1* trials served as the training set. For each combination of the ridge and time lag parameters (inner loops), a model was trained on the training set and tested on the held-out trial. This procedure was repeated for all λ values and time lag combinations (inner loops) across all trials (outer loops), resulting in performance metrics (i.e., correlation coefficients) for each parameter set across all folds. The optimal λ and time lag parameters were selected as those that yielded the highest average correlation across the outer folds." (Pages 34-35, Lines 22-8)

Regarding statistical tests for the encoding models, can you please add another sentence of detail to the "Monte Carlo method" used in the statistical test? Again, I suspect the lack of detail here is because the software is doing this all automagically, but it's hard to understand what's going on if you're not intimately familiar with the software itself. By "Monte Carlo", I assume you mean you're either randomizing the difference between two sets of model performance values, the permutation equivalent of a paired-samples t-test? And at other times you might be comparing the mean model performance to zero by flipping the sign of model performance values? Are these the randomizations? Second, I assume the software is using these randomizations to generate null clusters, and thresholding based on the size of clusters arising in the permuted data—is this correct? Please clarify.

The Monte Carlo method is a non-parametric randomization approach implemented in the FieldTrip toolbox. This method estimates statistical significance by generating a null distribution through repeated random permutations of the data. We applied this method to two types of comparisons: (1) comparing model performance values against zero (Figure 4 in the revised manuscript, previously Figure 3), and (2) comparing model performance between two models (i.e., testing whether the difference scores deviate from zero). In both cases, during each permutation, the data from the two conditions were either left unchanged or swapped (i.e., sign-flipped for the difference scores) within each participant. Group-level test statistics (i.e., t-values) were computed across all voxels for each permutation.

To correct for multiple comparisons, we used the cluster-based maxsum method. In this method, voxel-wise t-values were thresholded at a cluster-forming alpha level of 0.025 (i.e., 0.05/2 for two-sided testing). Spatially "adjacent" and statistically "significant" voxels were grouped into clusters, and each cluster was assigned a statistic equal to the sum of its t-values. Only the largest cluster-level statistic was stored for each permutation. We performed 1,000 permutations to form a null distribution of maximum cluster sums. An observed cluster was considered statistically

significant if its summed t-value exceeded the corresponding percentile of the null distribution (0.05/2 for two-sided testing). Accordingly, we have revised the manuscript as follows..

Methods section:

> "Specifically, the Monte Carlo method is a non-parametric randomization approach used to estimate statistical significance by generating a null distribution through repeated permutations of the data. In each permutation, the data from the two conditions (e.g., Model 1 performance vs. zero performance) were either left unchanged or sign-flipped within each participant to simulate the null hypothesis. Group-level test statistics (i.e., t-values) were then computed across all voxels for each permutation. To correct for multiple comparisons, we employed the cluster-based maxsum method. In the method, voxel-wise t-values were thresholded at a cluster-forming alpha level of 0.025 (0.05/2 for two-sided testing). Spatially, "adjacent" and "significant" voxels were grouped into clusters, and each cluster was assigned a statistic value equal to the sum of its t-values. Only the largest cluster-level statistic value was kept. We performed 1000 permutations to form a null distribution of maximum cluster sums. An observed cluster was considered statistically significant if its summed t-value exceeded the threshold percentile of the null distribution (0.05/2 for two-sided testing)." (Pages 36-37, Lines 18-8)

Page 32, line 1: "identify matrix" > "identity matrix"

We thank the reviewer and have updated the manuscript accordingly.

Reviewer #3: The authors provided a thorough revision, with new analyses and revision of the figures, as well as the text, which greatly improves the clarity of the manuscript and strengthens the message.
In my view, there are two main points to still be addressed:

We thank the reviewer for the careful evaluation and for highlighting the design question and the need for improved RT visualization.

1) I did not quite understand the new argumentation regarding the short and long foreperiods.

e.g. in the response to the respective comment:
" Initially, we categorized the foreperiod into two groups:
short (0.4 to 1.1 seconds) and long (1.3 to 2 seconds), primarily to easily explain the experimental design. However, we recognize that this categorization may suggest misleadingly that participants need to differentiate between the two categories to learn the conditional probabilities."

p. 7, l. 1-3:
"To simply explain the design in the
following, we categorized the foreperiods as short (between 0.4 and 1.1 seconds, denoted as S) or long (between 1.3 and 2 seconds, denoted as L). "

p. 7, l. 15:
"It is important to note that the conditional probability of FP2 is computed
based on each FP1 duration, as shown in Figure 1D (right column), rather than based on the short
or long labels."

If I do not completely misunderstand, the manipulation of predictability is that in some blocks, a 'short' foreperiod is followed by a short one, and in other blocks, more often by a 'long' one. The probability density functions in Figure 1 also seem to depict this. What did participants learn if not those distinctions?

We thank the reviewer for pointing this out. We now better understand the core of the concern. Combined with your previous question: *Additionally, the modulation of transition probabilities might have been somewhat weekend by drawing the individual foreprериods from uniform distributions with a relatively broad range: in some cases, a short FP of 1.1 s and a long one of 1.3 s were almost undistinguishable. Please motivate this choice*; the main concern is the small distinctions between 1.1-second and 1.3-second FP, which cause difficulty in learning conditional probabilities. We agree with you, and we had considered it during the design phase. Our motivation for still including these durations was to preserve a near-continuous distribution of foreperiods across the 0.4–2.0 second range (with a 0.1-second step and only 1.2-second FP removed), allowing more accurate computation of hazard functions over time. Specifically, as Supplementary Figure 10 shows, FPs in 1.1 and 1.3 seconds, as well as 0.4, 0.5, 0.6, 1.8, 1.9, and 2 seconds were assigned only one trial each. On the other hand, the main contributors were 0.7, 0.8, and 0.9 s (10, 25, and 10 trials respectively) (to the short category), and 1.5, 1.6, and 1.7 s

(10, 25, and 10 trials respectively) (to the long category). The less-informative durations (e.g., 1.1 and 1.3 s) were retained with only one trial each to maintain the probability distribution curve and also minimally impact the learning of conditional probabilities. As we can see from the HF, the value of these trials is close to zero. Its influence has now been verified by removing the 1.1- and 1.3-sec FPs in the analysis. The results shown below are similar to the main results (Table 3).

In addition, we randomly selected FPs to be a sequence pair. Thanks to Reviewer 1 (Reviewer 3 for you), we also computed hazard functions based on the actual probability distributions (Supplementary Figure 9) and demonstrated similar results with the modeled hazard functions (Supplementary Figure 13 & Supplementary Tables 10-12).

We thank the reviewer for pointing this out. We now better understand the core of your concern. Combined with your earlier comment:
"Additionally, the modulation of transition probabilities might have been somewhat weakened by drawing the individual foreperiods from uniform distributions with a relatively broad range: in some cases, a short FP of 1.1 s and a long one of 1.3 s were almost indistinguishable. Please motivate this choice."

The main concern relates to the small distinction between the 1.1-second and 1.3-second foreperiods, which could potentially cause difficulty in learning conditional probabilities. We agree with this point, and in fact, had considered it carefully during the design phase. Our motivation for still including these durations was to preserve a near-continuous distribution of foreperiods across the 0.4–2.0 second range (with 0.1-second steps and only the 1.2-second foreperiod removed). This design choice enabled more accurate computation of hazard functions over time. As shown in Supplementary Figure 10, the foreperiods at 1.1 and 1.3 seconds—as well as 0.4, 0.5, 0.6, 1.8, 1.9, and 2.0 seconds—were each assigned only one trial. In contrast, the primary contributors to the short and long categories were 0.7, 0.8, and 0.9 seconds (with 10, 25, and 10 trials respectively) for the short category, and 1.5, 1.6, and 1.7 seconds (also with 10, 25, and 10 trials) for the long category. The less-informative durations (e.g., 1.1 and 1.3 seconds) were retained with only few trials each in order to maintain the shape of the overall probability distribution while minimizing their influence on conditional probability learning. As reflected in the hazard function, the values of these trials were close to zero.

We have also confirmed their minimal impact by repeating the analysis with the 1.1- and 1.3-second foreperiods excluded. The results, shown below and in **Supplementary Table 13**, are consistent with our main findings (Table 3).

Supplementary Table 13. Effects of HF_U and HF_C on reaction times following FP2 (*1.1- and 1.3-sec FP removed*)

	Estimates	SE	β	*t value*	*p*	*Con R^2*
(Intercept)	0.244	0.007		34.93	<0.001	0. 222
HF_U	-0.050	0.005	-0.22	-10.52	<0.001	
HF_C	-0.009	0.002	-0.04	-4.08	<0.001	
HF_U * HF_C	0.042	0.005	0.19	8.10	<0.001	

n = 17439 observations.

Accordingly, we have added our motivation to the manuscript for clarification.

Methods section:

> "As shown in Supplementary Figure 10A, we assigned one trial each to foreperiods of 0.4, 0.5, 0.6, 1.0, 1.1, 1.3, 1.4, 1.9, and 2.0 seconds to maintain a nearly complete and continuous distribution of foreperiods within each block. This design allowed for more accurate estimation of hazard functions over time while minimally affecting the learning of conditional probabilities. In particular, foreperiods of 1.1 and 1.3 seconds—which are difficult to clearly categorize as short or long—were included with only one trial each. Their influence was confirmed to be minimal by control analyses excluding these foreperiods (Supplementary Table 13), which yielded results similar to the main findings (Table 3)." (Page 27, Lines 14-21)

2) I agree with Reviewer 3 that the presentation of results mainly through quantifications of model fits makes it hard for the reader to assess the actual explanatory power of the models. The revision provides convincing control analyses, but I think it would also be helpful to provide the display of reaction times in the main manuscript. Plotting the temporal response functions would also be helpful, at least in a supplementary figure.

We agree with the reviewer that our convincing results from additional and control analyses can be further strengthened by visualizing reaction times over time alongside the predicted values. Accordingly, we have added **Figure 2** and **Supplementary Figure 1** and plotted the temporal response functions at zero-second lag in **Supplementary Figures 3, 4, and 5.**
